# Quality management overview for the production of a tissue-engineered human skin substitute in Malaysia

**DOI:** 10.1186/s13287-023-03536-9

**Published:** 2023-10-20

**Authors:** Wan Tai Seet, Mohd Asyraf Mat Afandi, Mohamad Fikeri Ishak, Muhammad Najib Fathi Hassan, Nazeha Ahmat, Min Hwei Ng, Manira Maarof

**Affiliations:** Centre for Tissue Engineering and Regenerative Medicine, Faculty of Medicine, Universiti Kebangsaan Malaysia, Jalan Yaacob Latif, Bandar Tun Razak, 56000 Kuala Lumpur, Malaysia

**Keywords:** Quality assurance, Quality control, Quality management, Good manufacturing practice, Cleanroom, Tissue engineering, Skin substitute, MyDerm^®^

## Abstract

Treatments for skin injuries have recently advanced tremendously. Such treatments include allogeneic and xenogeneic transplants and skin substitutes such as tissue-engineered skin, cultured cells, and stem cells. The aim of this paper is to discuss the general overview of the quality assurance and quality control implemented in the manufacturing of cell and tissue product, with emphasis on our experience in the manufacturing of MyDerm^®^, an autologous bilayered human skin substitute. Manufacturing MyDerm^®^ requires multiple high-risk open manipulation steps, such as tissue processing, cell culture expansion, and skin construct formation. To ensure the safety and efficacy of this product, the good manufacturing practice (GMP) facility should establish a well-designed quality assurance and quality control (QA/QC) programme. Standard operating procedures (SOP) should be implemented to ensure that the manufacturing process is consistent and performed in a controlled manner. All starting materials, including tissue samples, culture media, reagents, and consumables must be verified and tested to confirm their safety, potency, and sterility. The final products should also undergo a QC testing series to guarantee product safety, efficacy, and overall quality. The aseptic techniques of cleanroom operators and the environmental conditions of the facility are also important, as they directly influence the manufacturing of good-quality products. Hence, personnel training and environmental monitoring are necessary to maintain GMP compliance. Furthermore, risk management implementation is another important aspect of QA/QC, as it is used to identify and determine the risk level and to perform risk assessments when necessary. Moreover, procedures for non-conformance reporting should be established to identify, investigate, and correct deviations that occur during manufacturing. This paper provides insight and an overview of the QA/QC aspect during MyDerm^®^ manufacturing in a GMP-compliant facility in the Centre for Tissue Engineering and Regenerative Medicine, Faculty of Medicine, Universiti Kebangsaan Malaysia.

## Introduction

In recent years, the development in cell therapy and stem cell research has led scientist to engage in the production of cells and tissue product that is of clinical grade. This can be achieved by following strict protocol of good manufacturing practice (GMP) to maintained product quality, safety and efficacy [1]. As GMPs revolve around process documentation, the quality of a product cannot be determined by examining or testing the final product alone [2]. GMP ensures that the quality of the final product, which focuses on manufacturing procedures, adheres to specifications and standards. Thus, based on current GMP principles, the quality of the starting and raw materials, and the procedures for production, sample collection, and cryopreservation should conform to organisational standard operating procedures (SOP) [3]. It is highly recommended to perform cell manufacturing for therapeutic use in a well-designed GMP-compliant cleanroom facility. Manufacturing such a product in accordance with GMP requirements will guarantee its safety, efficacy, and quality [4].

Quality assurance (QA) and QC are two subsets of quality management: QA is an overall management plan to guarantee the integrity of the product. QA is used to prevent errors and defects in the manufactured products. QA can take the form of SOP to manage the quality of raw materials, and production, maintenance, and inspection processes [5]. The other aspects of QA also include, but are not limited to, the implementation of non-conformance reporting (NCR), corrective and preventive actions (CAPA), management review meetings (MRM), vendor auditing, complaint, recall, process deviation management, risk management, change management, archiving system, batch record maintenance, calibration records, personnel training and assessment, equipment and facility qualifications, and logs [6]. On the other hand, QC is a part of quality management that focuses on inspecting, testing, and evaluating the manufactured product to assess its quality. QC checks manufacturing records and release criteria to ensure that the product has undergone required testing before it is released for use. Generally, QC is the process of identifying faults in the manufacturing process, where an NCR is generated if faults are found, and corrective actions are implemented [5, 7].

Advances for treating skin injuries have improved tremendously. These improvements have resulted in low mortality, shorter hospital stay, and reduced long-term morbidity [8]. Treatment of skin injuries typically involves the transplantation of autologous split skin grafts (SSG). Having been developed thousands of years ago, split skin grafting has been the mainstay procedure and is considered the gold standard for treating skin injuries, especially for burn wound coverage [9]. Nonetheless, the use of SSG is accompanied by its own set of challenges. This is particularly evident in the case of donor site absence, where a limited amount of healthy native skin can be used, which can eventually lead to poor coverage of the autologous skin graft to the patient [9]. Another issue that can arise from SSG use is donor site morbidity, which has attracted attention recently. Although SSG have many advantages, the skin harvesting creates secondary injuries. Such injuries can lead to numerous other problems, which include infections and hyperpigmentation, and prolonged pain in some patients [10]. Thus, researchers and clinicians have suggested numerous alternatives to treat skin-related injuries, such as allogeneic and xenogeneic transplants [11], or with recent alternatives, by using cultured cells, stem cells [12], and dermal substitutes such as tissue-engineered skin [13].

Recently, cell therapy has been widely used and accepted in skin injury treatment plans, especially extensive burns. Cell therapy can take the form of keratinocyte suspensions or sheets, or dermal–epidermal skin substitutes [14, 15]. Thus, to manufacture GMP-grade cells for therapeutic usage and to facilitate the production of MyDerm^®^, an autologous skin substitute intended for treating diabetic ulcers, burns, and traumatic injury, we designed and established a cleanroom facility affiliated with the Centre for Tissue Engineering and Regenerative Medicine (CTERM), Faculty of Medicine, Universiti Kebangsaan Malaysia. This study discusses the QA and QC for the GMP manufacturing of MyDerm®, which comprises procedures that range from the materials selection criteria, quality risk management, process validation, and the in-process QC and release criteria.

## Materials and methods

### Selection of materials

Materials include all components, materials, or supplies to be incorporated or used in the manufacture of the product. The Pharmaceutical Inspection Co-operation Scheme (PIC/S) states that the source, origin, and suitability of all starting materials should be clearly defined [16]. Whenever possible, research-grade reagents should be substituted with the appropriate reagents of GMP, clinical, or ‘for further manufacturing’ grade in the manufacture of a product intended for clinical use. Many research-grade reagents contain animal-derived products that may increase the risk of transmissible spongiform encephalopathy (TSE) [17]. Many people have immunoglobulin E (IgE) antibodies against bovine serum albumin (BSA) that will lead to the development of anaphylactic reactions [18]. Therefore, it is important to exclude or substitute components suspected to have adverse effects on humans, such as the capacity to cause an immune response or to act as carriers of transmittable diseases [19].

During the manufacture of MyDerm^®^, skin cells are harvested with collagenase type I (Worthington, NJ, USA) and recombinant trypsin–EDTA (TE) solution (Biological Industries, CT, USA) [14]. Both enzymes are derived from bacterial sources. In MyDerm^®^ production, the cell culture medium for human dermal fibroblasts is supplemented with autologous human serum instead of foetal bovine serum (FBS) to eliminate the risk of TSE and the immune response. Human serum has been proven to be a more advantageous supplement compared with FBS in terms of cell expanding capability and gene expression of the extracellular matrix proteins involved in wound healing [20]. Recently, researchers worldwide also explored the use of human AB serum [21], plasma-rich platelets [22], platelet lysate [23], or other FBS alternatives [24] given ethical concerns, supply shortage, increased pricing, and FBS fraud [25–28].

TE is a common enzyme used to disassociate cells during cell culturing. However, most TE solutions are research-grade and derived from porcine or bovine pancreas. The use of animal-derived enzymes may also raise religious concerns. Furthermore, animal-derived TE is harsh and can cause cellular damage. TE is required for initial tissue digestion during MyDerm^®^ production, as a stronger enzyme is needed to harvest the cells from the tissue. As animal-derived enzymes are not recommended, a recombinant TE is used as an alternative [29]. The recombinant TE is subsequently replaced with TrypLE™ Select (Gibco, NY, USA), which is gentler on cells and can minimise cellular damage. TrypLE™ Select is also a GMP-grade trypsin produced using recombinant technology that is free from animal derivatives, hence eliminating any religious concerns [29]. Unlike TE, which must be stored at − 20 °C, TrypLE™ Select is stable at room temperature or 2–8 °C, making it easier to use and preventing the loss of functionality due to repeated freezing and thawing.

During the initial cell culture process at passage 0, bacterial contamination is prevented by antibiotics such as penicillin–streptomycin–amphotericin B and gentamicin. These antibiotics are GMP- and clinical-grade, respectively, and undergo full safety testing with the aim of minimising the probability of adverse events. These antibiotics have more extensive qualifying documentation than research-grade reagents to meet the required purity and quality. All culture media and reagents used are also of for-further-manufacturing-grade, except for EpiLife^®^ medium (Gibco), which is a research-grade reagent used for human keratinocyte growth. Research-grade material can be used when a higher-grade material cannot be identified, but a risk assessment must be performed to ensure that the residual material remaining in the final product is adequately removed and the risk of transferring the material to the recipient is minimal [30].

### Management of materials

Starting materials are crucial in the manufacture of biological products. The management of these procedures is necessary to regulate all materials used in GMP facilities [31].

### Materials specification (MS) and register

All materials must have a documented MS, which includes but is not limited to a brief description of the intended use, packaging, storage conditions, shelf-life requirements, occupational health and safety considerations, testing requirements, and acceptance criteria. The materials along with their product codes, manufacturer, and vendor’s contact are recorded in the materials register to control the materials ordered, vendors, and manufacturers. The relevant MS and materials register must be updated in the event of a change to a specific material or vendor.

### Receipt and processing of materials

Upon delivery, all materials must be examined, and a materials receipt and verification record must be created in accordance with the applicable CMS [32]. The packing must be examined and it should be verified that the outer packaging has no significant visible damage. The delivery is accepted based on inspection of the outer packaging and then ensuring that the inner packaging is intact. Upon completion of the delivery inspection, if the goods cannot be inspected or require further testing or verification, a QUARANTINED label must be applied to the outer primary container, and the items must be stored in a quarantined area until they can be examined or tested.

If the items delivered do not fulfil the acceptance criteria listed in the MS, they should be rejected, and a REJECTED label must be attached. The material must be stored separately from the products released to avoid accidental use, and the vendor should be contacted for further discussion. A material defect report will be filled in to detail the reason for rejection, and the organisation can request for corrective actions or a replacement from the vendor.

If the delivery passes the inspection and verification, a RELEASED label is attached to the items and the items will be approved for cleanroom use. All released items should be relocated to their proper storage places inside the cleanroom according to the manufacturers’ recommendations.

### Storage and monitoring

All materials must be stored in accordance with the manufacturers’ specifications. Before use, the item expiration date must be verified. Stock must be managed to ensure that the products with the shortest expiration date are used first. All expired inventories must be marked as REJECTED and removed from circulation. The temperature should be monitored in all storage rooms for materials. The materials should be quarantined if the storage conditions are outside the specified acceptable ranges. A QUARANTINED label should be attached to the item until a written confirmation is obtained from the manufacturer/vendor stating that the item is still fit for use.

### Quality risk management

Quality risk management is a systematic process for the assessment, control, communication, and review of risks to the quality of the product [16]. Risk management begins with the identification of risk and recognising and describing the risk, followed by an analysis to determine the nature and level of the risk. The risk assessment results will determine if the risk is acceptable or requires mitigation. The three possible risk assessment outcomes include: low risk (acceptable risk with no action), medium risk (risk mitigation required), and high risk (unacceptable risk and significant changes are required). Risk level can be analysed and calculated using the risk matrixes shown in Tables 1, 2 and 3 [33].

**Table 1:** Probability of an Occurrence

Score	Probability	Description
5	Most Certain	Events are expected to occur or will occur periodically with high probability. More than once in a year
4	Likely	Events have a probability of happening. Once in 1–3 years
3	Possible	Events are likely to occur. Once in 3–5 years
2	Unlikely	Events can happen. Once in 5–10 years
1	Rare	Events are not likely to occur or will only occur in exceptional circumstances. Once in 10 or more years

**Table 2:** Impact of hazard

Score	Impact	Example detail description
1	Insignificant	No injuries, low financial loss, no risk to reputation
2	Minor	Minor First aid treatment, on-site release immediately contained, medium financial loss, some customer dissatisfaction
3	Moderate	Medical treatment required, on-site release contained with outside assistance, high financial loss and public visibility
4	Major	Major Extensive injuries, loss of production capability, invocation of disaster recovery with no detrimental effects, major financial loss
5	Catastrophic	Death, off-site with detrimental effect, huge financial loss

**Table 3:** Level of risk

Probability (P)		Impact (I)				
		1	2	3	4	5
5		5	10	15	20	25
4		4	8	12	16	20
3		3	6	9	12	15
2		2	4	6	8	10
1		1	2	3	4	5

Risk	Level	Actions				
15–25	High	Immediate action required to control the incident				
5–12	Medium	Control the incident via planned approach and apply temporary measures				
1–4	Low	May be acceptable and further reduction may not be necessary				

*P* × *I* = Relative Risk

*P* = Probability

*I* = Impact

One risk assessment when manufacturing MyDerm^®^ is dilution study for the removal of research-grade EpiLife^®^ medium and human keratinocyte growth supplements (HKGS) from the final product. As higher-grade (for further manufacturing- or clinical-grade) EpiLife^®^ medium and HKGS are not available in the local market, the remnant medium and growth supplements volume should be kept as low as possible in the final product to minimise the risk of introducing a research-grade material to the patient. Another risk assessment is the spiking test on antibiotic-free culture medium. During the first few days, the cells are cultured with culture medium containing antibiotics to prevent contamination induced by the skin biopsy. The antibiotics will be removed from the culture medium after the first trypsinization. The spent antibiotic-free culture medium will undergo sterility testing after a few days as in-process QC. To ensure that residual antibiotics do not affect the negative results obtained, the culture medium is intentionally spiked with various bacteria and fungus to ensure that it can support microorganism growth. A dilution study is also performed to ensure that the amount of antibiotics remaining in the final product is insignificant and unlikely to pose any danger to the recipient. Risk management is an important aspect, as uncontrolled risks can cause damaging effects such as financial loss, bad reputation, and even legal issues for an organisation.

### Vendor selection

A vendor is anyone who provides goods or services to a company or individuals. Vendors of our facility were selected based on their ability to consistently meet specifications for incoming raw materials, deliver materials punctually, provide quality products at a reasonable cost, and their commitment to customer service and technical support. They will be requested to answer a series of questions, and will be qualified or disqualified as approved vendors according to the outcome of the answers provided. Approved vendors will also undergo yearly user evaluation based on their performance. The score obtained must be above the cut-off point determined by the organisation before they can be re-listed as approved vendors the following year.

### Validation

**Fig. 1:**
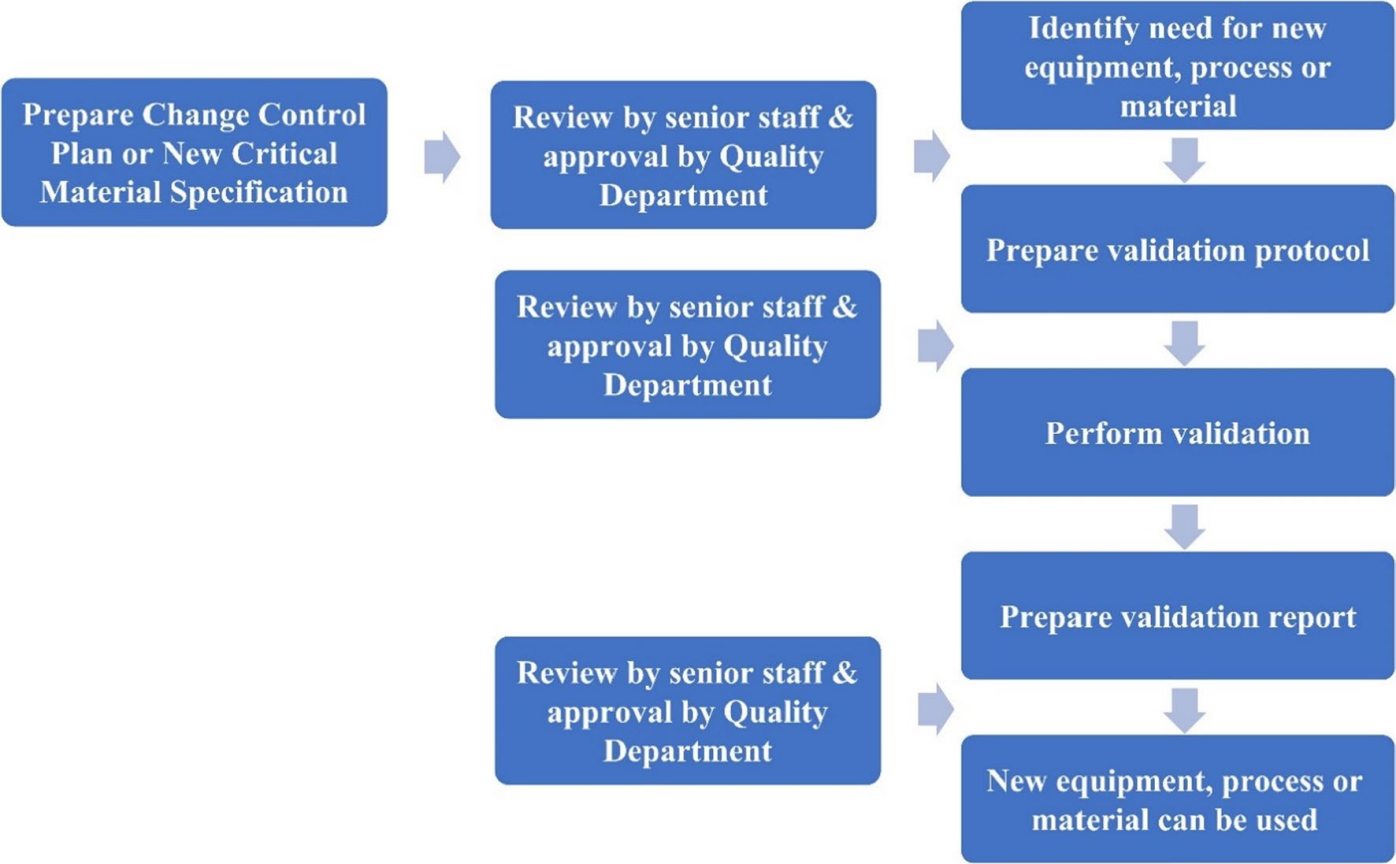
Flow chart of validation process. Process validation ensures manufacturing process produces quality products

Validation involves verifying the materials, procedures, activities, systems, or equipment used during production to control and produce products with consistent specifications. Identifying new procedures, equipment, or materials to be introduced into the quality system is the initial step in validation. A validation activity is conducted following the preparation of the validation methodology. The report is prepared, and the new procedures, equipment, and/or materials will be ready for use upon revision by senior staff and Quality Department approval (Fig. 1).

#### Label validation

**Fig. 2:**
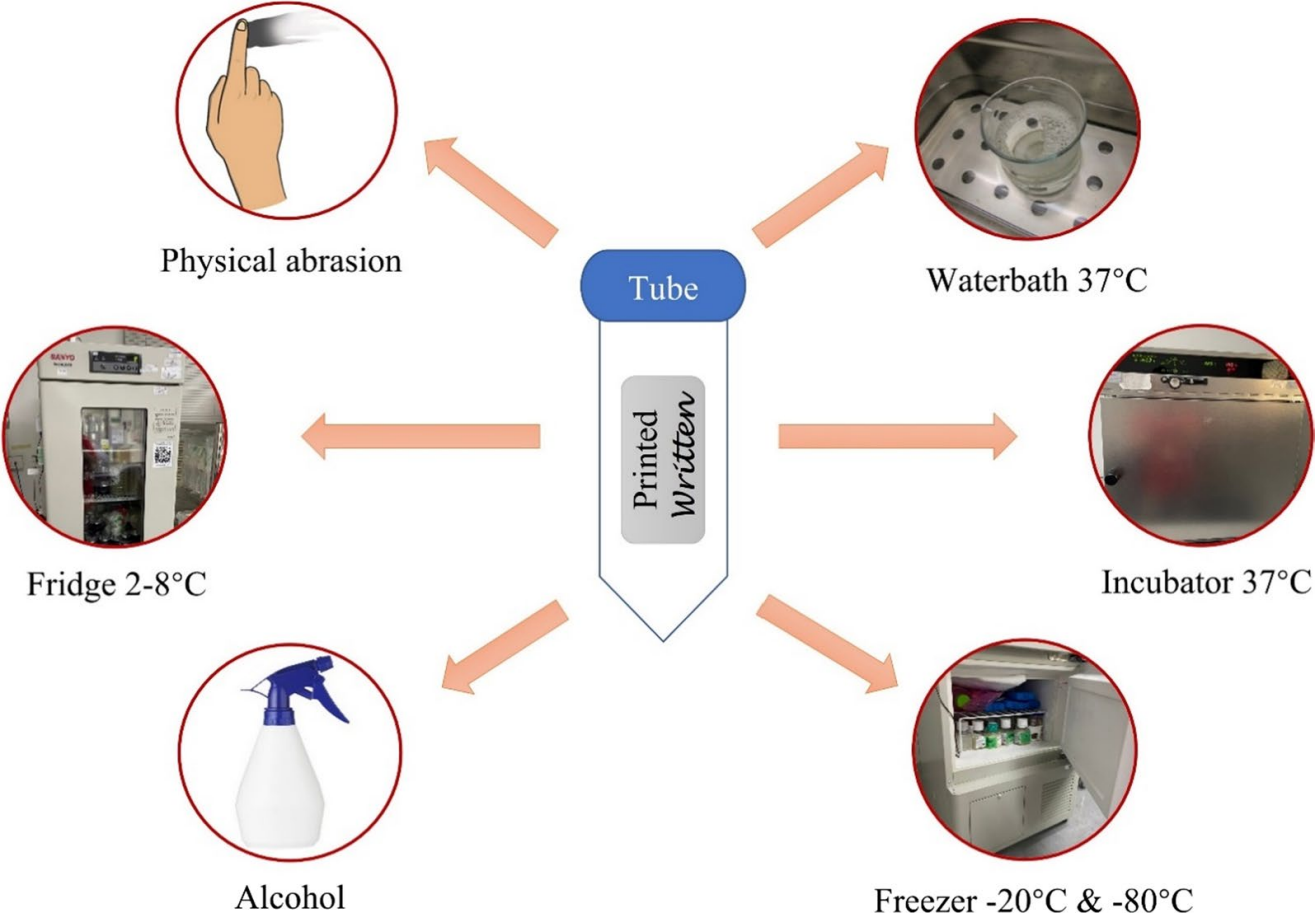
Printed and handwritten labels are subjected to different conditions, mimicking manufacturing processes

Labels are used to identify starting materials and intermediate and finished products, and are important for product traceability. Therefore, it is necessary to validate the suitability of the labels for use during manufacturing [34]. Printed and handwritten labels are subjected to conditions such as different storage temperatures, water immersion, alcohol exposure, and physical abrasion to mimic the actual manufacturing process (Fig. 2). This process also validates the printing techniques (e.g. laser printing or thermal transfer) and the brand of marker pen used. The labels are checked for adhesiveness, readability, and weathering at the end of the validation activity and are considered acceptable if they pass the validation. The validation should be repeated if a new brand of label, printing technique, or marker pen is introduced.

#### Operator aseptic validation

Operator aseptic validation is the procedure necessary to understand the aseptic technique and the requirements before engaging in aseptic processing of intermediate and final products [35]. All operators must pass an operator-related proficiency assessment before they can work independently in the cleanroom facility. At CTERM, the operator aseptic validation is performed once every 2 years. Operators’ gowning techniques are continuously monitored via personnel micro-surveillance, where finger dabs and gown swabs are performed on TSA plates after operators have performed the cleanroom gowning procedure. This personnel micro-surveillance monitoring is performed once every 2 months to ensure that operators maintain proper aseptic technique competence to prevent the introduction of contamination into the facility and to ensure control over product sterility and safety [36]. Operators must also perform finger dabs after completing every manufacturing process. Furthermore, all operators must undergo proper aseptic technique training and assessment, including hand-washing, open donning, and cleanroom garment gowning before entering the cleanroom facility.

#### Transport validation

The Good Distribution Practice (GDP) guidelines published by Ramli Zainal from the National Pharmaceutical Regulatory Agency (NPRA) states that materials or products should be stored and transported under controlled circumstances to prevent mishaps that could affect product specificity [37]. The product packaging must be able to safeguard against contamination, spills, and breakage. The finished products should be packed in a biosafety cabinet to preserve sterility. For temperature-sensitive products, ice packs are shipped together in the shipping container to maintain the temperature, and temperatures during transportation are recorded using a temperature recorder. Product information must also be included as suggested by PIC/S and the World Health Organisation (WHO) to enable product traceability [38].

Transporting products requires committed workers who are well-trained and sufficiently knowledgeable to deliver the goods on schedule. Worst-case scenarios should be considered, all potential delivery routes should be planned, and the temperature should be monitored during shipment. At CTERM, the MyDerm^®^ construct is transported a few floors down to the operating theater for our clinical studies. The product is expected to be hand-carried and transported downstairs by an elevator. During worst-case scenarios, the personnel might need to transport the product by using the stairs. Therefore, transport validation is performed to ensure that the temperature can be maintained during transportation based on a worse-case scenario. As the construct is stored in a non-leak proof OmniTray*™* (Nunc, MA, USA), the tray must be sealed in double-layer sterile plastic bags to ensure product sterility during transport. Leakage validation should be performed on the packaging to ensure that the product integrity is maintained.

#### Use testing

Use testing is performed to validate reagents upon receipt to ensure that they are of sufficient quality to be used in the manufacturing process in a GMP facility. The use test is conducted on representative samples of the same-batch delivery to determine whether their primary function remains intact [18]. The reagents that require use testing by CTERM for MyDerm^®^ construction are: (a) digestion activity of enzymes for tissue specimens, (b) functionality of cell culture medium and growth supplements, (c) dissociation activity of enzymes for adherent cells, and (d) plasma polymerization activity for construct formation. After undergoing and fulfilling the use test release criteria, the validated reagents and plasma can be released for manufacturing use.

### In-process quality controls

#### Cell manufacturing process and performance

QC should be performed at appropriate stages during production to ensure the consistency of the product quality. QC involves testing units and determining if the final product is within specification or if there is a need for corrective actions in the manufacturing process [39]. As the final product cannot be sterilised, strict in-process control should be conducted to assure product quality and safety. The first evaluation aspect is to ensure that samples are not contaminated with bacteria and fungus. Such contamination is easily detected by the naked eye when the cell culture medium turns cloudy. The contamination of blood serum and plasma, which are used as cell culture medium supplement and biomaterial for MyDerm^®^ construction, respectively, is detected using the BD BACTEC™ automated blood culture system (BD, NJ, USA). Another quality control test is sterility testing of prepared cell culture media, as culture media preparation involves open processes that are at risk of contamination. Spent cell culture media are also tested to ensure that the sterility of the process is maintained. Contaminated cells not only affect the quality but also the safety of the end product. Therefore, the manufacturing process must be terminated immediately if contamination is detected.

Cell count and cell confluency are also considered in cell culture manufacturing. Determining the correct time to subculture cells can aid the maintenance of cell health and quality. Cells are best sub-cultured at the exponential phase. Performing trypsinization too early will waste materials, while delayed trypsinization causes cells to enter the stationary phase, or worse, the death phase. Late trypsinization might also cause slow cell proliferation in the next passage and lead to cell senescence [40]. In our practice, we trypsinize cells at 70% confluence, when most of the cells are still undergoing rapid proliferation.

Manufacturing processes control is also essential in a GMP setting. Batch manufacturing records, which combine procedures and forms, document every piece of processing information, which includes but is not limited to the batch number of the product being manufactured, the amount and lot number of the reagent/medium used, the process start and end date, the timing of critical steps, the initials of the operator performing critical processes, cleanroom certification, and equipment calibration status. The batch manufacturing record is a critical record that ensures that the product achieves the desired quality and adheres to regulatory requirements [41]. Batch manufacturing record contents and formats vary between companies and product requirements.

#### Environmental monitoring (EM)

**Fig. 3:**
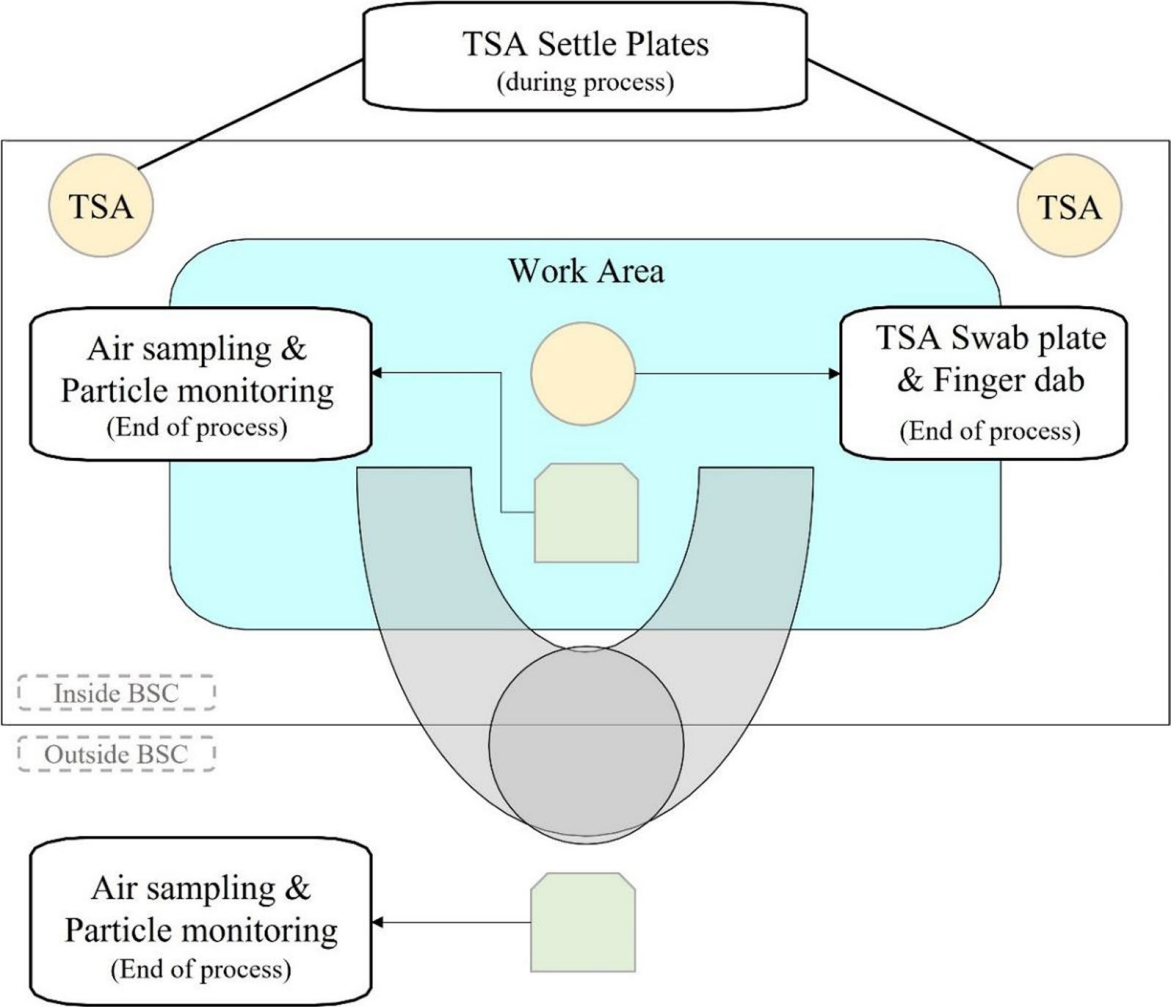
Schematic of in-process environmental monitoring locations within the BSC

In-process EM is another important aspect in GMP manufacturing to monitor operator aseptic technique and environment cleanliness. During open manipulation, two tryptic soy agar (TSA) plates are placed in the biosafety cabinet (BSC) as settle plates to assess the likelihood of microorganism deposition into the cell culture. The two settle plates are changed every 2 h to prevent the plates from drying out, which would lead to inaccurate in-process EM results. The BSC work area is also swabbed at process completion, demonstrating that the BSC remains in ‘clean’ condition while open manipulation is performed. Air sampling and particle count inside and outside the BSC are also performed at the end of the production process to ensure that the cleanliness is within specifications (Table 4). Figure 3 illustrates the overall in-process EM during open manipulation. All results and observations obtained during the in-process EM are recorded as near as is practical to the time of the occurrence. Whenever an out-of-specification event (Table 5) or procedure non-conformance as outlined in the SOP occurs, the operator must raise an NCR as soon as is practical.

**Table 4:** Maximum limit for particle count and air sampling for in process environmental monitoring

	0.5 um	5.0 um
*Maximum allowable particle count during in process event (particles/m*^*3*^*)*		
Grade A (Inside BSC)	3520	20
Grade B (Outside BSC)	352,000	2900
*Maximum allowable CFU/plate for air sampling*		
Grade A (Inside BSC)	< 1	
Grade B (Outside BSC)	10

**Table 5:** Alert and action limit of CFU per plate for in-process environmental monitoring

Environmental monitoring	CFU alert limit	CFU action limit
Gown swabs	≥ 2 per plate	≥ 5 per plate
Finger dabs	≥ 2 per plate	≥ 5 per plate
Finger dabs—at the end of process	N/A	> 1 per plate
In process settle plate EM	N/A	> 1 per plate
In process swab plate EM	N/A	> 1 per plate

#### Non-conformance report

An NCR is a document that identifies and reports divergences between the actual condition of a product, service, or process and the requirements defined by quality standards, which include but are not limited to the PIC/S guideline, SOP, and material specifications. The issue can be defined, analysed, and corrected accordingly, and the NCR ensures that the non-conformities are addressed and that the end product safety and quality are met. Furthermore, the NCR aids the creation of a regulatory plan of action to prevent the recurrence of divergences and assists further compliance or audits. The NCR information differs between organisations, as the NCR can be customised to fit specific organisation requirements. Examples of typical information in an NCR are the date of the incident, date of the non-conformance identification, NCR classification and code, the product affected (if any), the description of the incident, root cause, and proposed CAPA. Generally, non-conformance can be classified as critical or non-critical based on the severity and effect on the end product. All critical NCRs are closed out before the release of the associated product, while all non-critical NCRs must demonstrate acceptable progress at the time of release of an associated product.

#### Release criteria

The release criteria are established and act in accordance based on the product to ensure the identity, safety, purity, and potency of the final product before it is released [42]. Generally, product release criteria are separated into product physical characteristics and sterility testing. Table 6 depicts some release criteria for MyDerm^®^ that must be fulfilled before the product can be released. Other important criteria that can be included to ensure that the standard quality of the cell- and tissue-based final products are within the acceptable release criteria include biocompatibility, genotyping, karyotyping, and cell characterisation analysis.

**Table 6:** Release criteria for MyDerm^®^

Test	Specification
*Physical characteristics*	
Viability	≥ 70%
Total number of keratinocyte passages	≤ 6
Keratinocyte cell count	up to 2 × 10^7^ (depending on size)
Total number of fibroblast passages	≤ 6
Fibroblast cell count	up to 2 × 10^7^ (depending on size)
Product released	≤ 72 h from end of manufacture
*Sterility testing*	
Micro-contamination testing	Not Detected
Mycoplasma testing	Not Detected
Endotoxin testing	< 0.25 EU/ml
Gram stain	No organisms detected

The other part of the release criteria is based on sterility testing results. Various factors might cause contamination at any stage of the manufacturing process, such as during media preparation, open manipulation, packaging, storage, or even during transportation [43]. The sterility test applied to the final product is considered a final series of QA/QC by which the sterility is assured [17]. For sterility testing, the cultured media of the cells during the last passage before constructs are tested for micro-contamination, mycoplasma, and endotoxin. Gram staining is performed on the day of release as a rapid microbial detection test, as the full micro-contamination result (14 days) might not be available at product release [44].

The absence of microbial contamination is an absolute measure of product safety. On completion of the manufacturing batch, a portion of the final product will undergo microbial contamination testing, which might involve several methods according to the product to be tested. These methods involve classical culture, biochemically based enumeration, modified culture, and microscopy-based enumeration [45]. For cell-based products, sterility testing of microbial contamination is conducted in accordance with the British Pharmacopeia 2013 guideline [46]. MyDerm^®^ is considered free from contamination if no growth is detected in the Day 3 interim result and is deemed fit to be released upon the fulfilment of other criteria. Nevertheless, the full 14-day results must be documented, and product recall must be initiated if growth is detected.

Mycoplasma is a gram-negative bacteria that is among the smallest free-living microorganisms, and is commonly found in mammals, plants, fish, reptiles, and humans. Mycoplasma lacks cell walls around the cell membrane, which renders it resistant to beta-lactam antibiotics. Mycoplasma can contaminate cell cultures due to mishandling or following transfer from harvested tissue samples. Due to its reduced metabolic rate and long generation time, it is difficult to detect mycoplasma via microscopic observation [47].

Mycoplasma infection can damage health and is potentially life-threatening. One of the leading morbidities and mortalities worldwide, community-acquired pneumonia is caused by *Mycoplasma pneumoniae* [48]. Hence, cell culture supernatants should be tested for mycoplasma contamination before product release to confirm that they are free from the bacteria [47, 49]. Several methods can be used to detect mycoplasma, such as direct microbial culture, deoxyribonucleic acid (DNA) staining, enzyme-linked immunosorbent assay (ELISA), and conventional and real-time polymerase chain reaction (PCR) assay.

Bacterial endotoxin testing is often used as a real-time marker of microbial contamination and testing is recommended especially when production involves the usage of water where the water sources, water treatment equipment, and treated water used might cause chemical or biological contamination and indirectly cause the contamination of the product [16]. Also known as lipopolysaccharides, endotoxins are the major component of the outer cell wall of gram-negative bacteria that are released after cell death and lysis. High endotoxin levels can cause septic shock, hypotension, and coagulopathies and can even lead to death [50, 51]. Endotoxin can be detected using the Limulus amoebocyte lysate (LAL) test, which is the most popular endotoxin detection technique used in pharmaceutical products [52]. The three common LAL tests used worldwide are the gel clot method, turbidimetric assay, and chromogenic assay. Endotoxin in MyDerm^®^ storage medium is detected using the gel clot method, which is the simplest and most economical LAL test. However, it is only a qualitative test. If quantitative results are required, a kinetic turbidimetric or kinetic chromogenic detection method can be used [53–55].

The acceptable endotoxin levels might differ between products. According to the US Food and Drug Administration (FDA), the limit of endotoxin for medical devices, which have direct contact with the cardiovascular and lymphatic systems, is 0.5 endotoxin units (EU)/ml or 20 EU/device. For devices that have direct contact with cerebrospinal fluid, the endotoxin level is limited to 0.06 EU/mL or 2.15 EU/device [56]. For MyDerm^®^ storage media, the endotoxin level limit is set at 0.25 EU/ml, which is similar to the allowable limits for water for injection, sterile water for injection, and sterile water for irrigation [57].

**Fig. 4:**
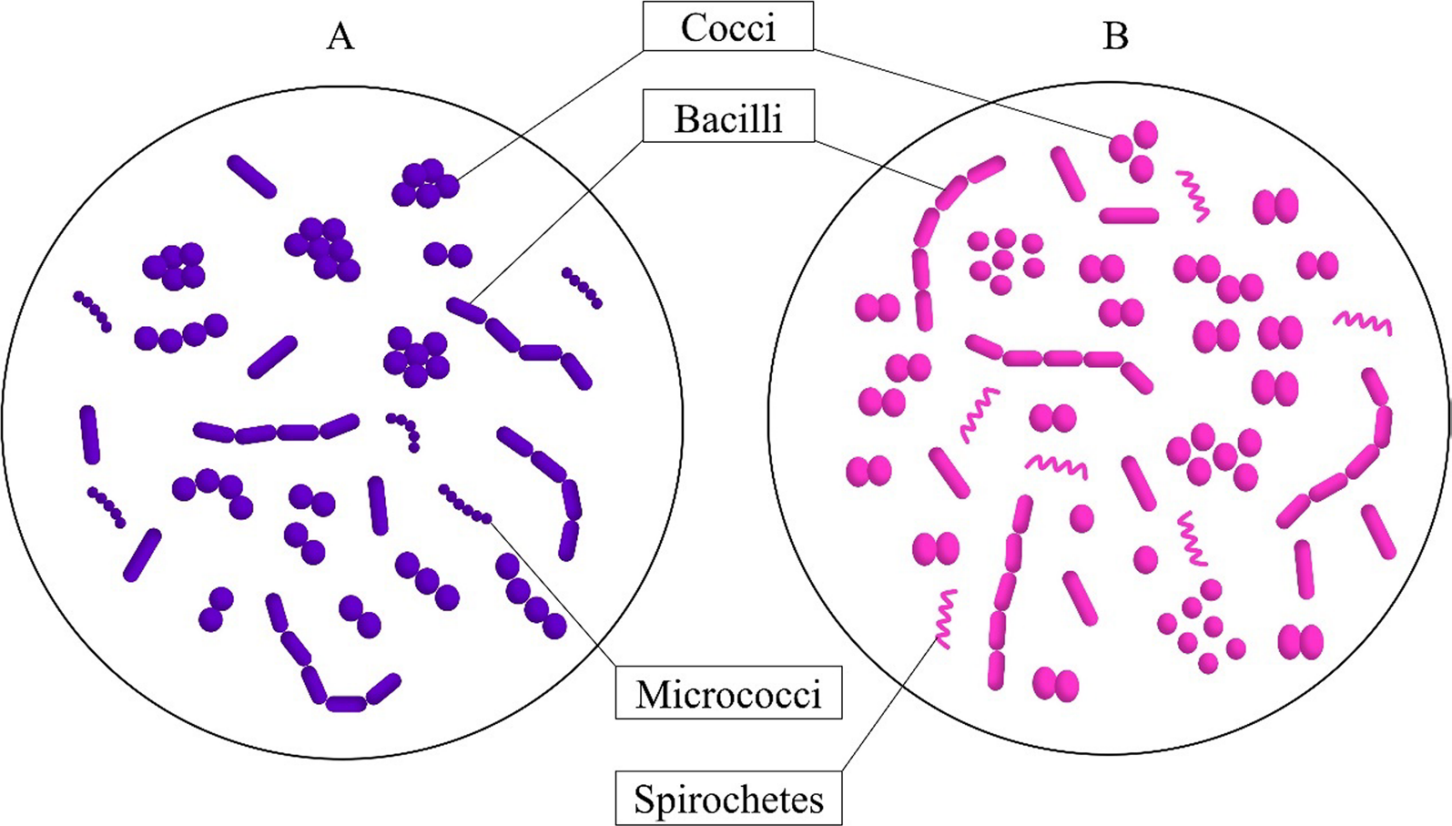
**A**) Gram-positive bacteria will stain dark blue/violet and **B**) Gram-negative bacteria will stain red/pink

Gram staining is used for several purposes, such as to provide preliminary information on presumptive bacterial pathogens, identify the bacteria types, and directly conduct microbiologic examination [58]. The Gram stain outcome identifies two different fundamental cell varieties based on colour: dark blue or violet (by crystal violet stain) indicate gram-positive bacteria, while red or pink (carbol fuchsin) indicate gram-negative bacteria, as illustrated in Fig. 4. As the Gram staining method is a qualitative data measurement, its sensitivity means that false positive results are possible. Limits such as < 5 or < 10 organisms per oil immersion field can be defined for some cell-engineered products. Thus, a laboratory that uses the Gram stain as a release criterion should specify the acceptability limit, which is the number of organisms for each product [59].

The Gram stain is used as a rapid micro-contamination detection test on the day of product release for cell-based products with short shelf-life when the 14-day micro-contamination test result is not yet available [44]. MyDerm^®^ has a short shelf-life of 72 h when stored in medium at 4 °C [13]. Typically, only the day 3 interim micro-contamination test result will be available on the day of product release. Hence, the Gram stain result coupled with day 3 interim results will be used as the release criteria.

As each product has differing product release criteria, scientific guidelines from previous or similar products are needed when developing the release criteria. The release criteria could also depend on the current regulatory requirements.

## Conclusion

In Malaysia, the NPRA regulates the manufacturing of cell and tissue products for therapeutic purposes. With the enforcement of Cell and Gene Therapy Product (CGTP) Guidelines in July 2021, we consider it crucial for a GMP facility to function and operate in accordance with current guidelines. The QA and QC practices at each step and process, from initial production until the release of MyDerm^®^, are in accordance with GMP standards, which will eventually deliver a product of the highest international standards. Thus, the manufactured cells are considered the most appropriate treatment option for cellular therapy. Although the process and steps were carefully designed and fine-tuned for manufacturing MyDerm^®^, we believe that the process described here can be translated and implemented in various settings to maintain the quality, safety, and reliability of a manufactured product.

## Data Availability

Not applicable.

## Abbreviations

BSA: Bovine serum albumin
BSC: Biosafety cabinet
CAPA: Corrective and preventive actions
CFU: Colony-forming unit
CGTP: Cell and gene therapy product
CO_2_: Carbon dioxide
CTERM: Centre for Tissue Engineering and Regenerative Medicine
DNA: Deoxyribonucleic acid
ELISA: Enzyme-linked immunosorbent assay
EM: Environmental monitoring
EU/ml: Endotoxin units per millilitre
FBS: Foetal bovine serum
FDA: Food and Drug Administration
GDP: Good distribution practice
GMP: Good manufacturing practice
HKGS: Human keratinocyte growth supplements
IgE: Immunoglobulin E
ISO: International Organisation for Standardisation
LAL: Limulus amoebocyte lysate
MRM: Management review meetings
MS: Materials specification
NCR: Non-conformance report
NPRA: National Pharmaceutical Regulatory Agency
PCR: Polymerase chain reaction
PIC/S: Pharmaceutical Inspection Co-operation Scheme
QA: Quality assurance
QC: Quality control
QMS: Quality management system
SOP: Standard operating procedure
SSG: Split skin graft
TE: Trypsin–EDTA
TSA: Tryptic soy agar
TSE: Transmissible spongiform encephalopathy
UKM: Universiti Kebangsaan Malaysia (National University of Malaysia)
WHO: World Health Organisation
